# Supplementary material for: Effect of intravenous vitamin C administration on chemotherapy-induced adverse events in patients with nasopharyngeal cancer
Source: PLoS One. 2026 Apr 13;21(4):e0346818. doi: 10.1371/journal.pone.0346818 (PMC13075686; doi:10.1371/journal.pone.0346818)
Supplement: Attachment — Submitted filename: Response to Reviewers.docx [file pone.0346818.s002.docx]

Dear Editor,

We would like to sincerely thank you and the reviewers for the insightful and constructive comments provided on our manuscript. We have carefully addressed all comments from the reviewers and revised the manuscript accordingly. A point-by-point response to each reviewer’s comments is provided below, with the corresponding changes made to the manuscript highlighted. We appreciate the opportunity to revise our work and hope that the revised manuscript will now meet the journal's standards.

Thank you for your consideration.

Sincerely,

Manupol Tangthongkum, MD

(Corresponding author)

**Response to Journal Requirement**

**Journal Requirement 1.** your reasons for your delay in registering this study (after enrolment of participants started);

**Journal Requirement 2.** confirmation that all related trials are registered by stating: “The authors confirm that all ongoing and related trials for this drug/intervention are registered”.

### ****Response:****

We sincerely thank the Editor for raising this important issue regarding trial registration.

The study was submitted to the Thai Clinical Trial Registry (TCTR) for registration at the time of study initiation. During the registry review process, the TCTR requested additional clarifications prior to granting final approval and assigning the registration number (TCTR20190316003). As a result of this administrative review process, the official registration date appears after enrollment of the first participant.

Importantly, institutional ethics approval had been obtained from the Ethics Committee of the Faculty of Medicine, Prince of Songkla University (REC.61-416-13-1) before participant enrollment began. The study protocol, eligibility criteria, predefined endpoints, and statistical analysis plan were finalized prior to enrollment of the first participant, and no methodological modifications were made between study initiation and trial registration.

The authors confirm that all ongoing and related trials for this intervention are registered.

Revised Materials and methods (page 6, paragraph 1, lines 121–126)

**Study design**

This prospective, randomized, double-blind, placebo-controlled trial was conducted at Prince of Songkla University, Thailand, between March 14, 2019 and December 20, 2020. The study protocol was approved by the Ethics Committee of the Faculty of Medicine, Prince of Songkla University (REC.61-416-13-1) and registered with the Thai Clinical Trial Registry (TCTR20190316003) on March 16, 2019. The study was submitted to the Thai Clinical Trial Registry at study initiation; however, due to administrative review and clarification requests, the official registration date appears after enrollment began. Ethics approval and the finalized protocol were completed prior to participant enrollment. The authors confirm that all ongoing and related trials for this intervention are registered. The study protocol adhered to relevant guidelines and regulations, including the World Medical Association Declaration of Helsinki on ethical principles for medical research involving human participants.

**Journal Requirement 4.** Thank you for stating the following financial disclosure:

“This research was financially supported by a grant from the Faculty of Medicine, Prince of Songkla University, Thailand.”

Please state what role the funders took in the study.  If the funders had no role, please state: "The funders had no role in study design, data collection and analysis, decision to publish, or preparation of the manuscript."

Please include this amended Role of Funder statement in your cover letter; we will change the online submission form on your behalf.

### ****Response:****

We thank the Editor for this important request for clarification.

The Faculty of Medicine, Prince of Songkla University, Thailand, provided financial support in the form of a research grant. **The funders had no role in study design, data collection and analysis, decision to publish, or preparation of the manuscript.**

We have amended the Role of Funder statement accordingly and will include the revised statement in the cover letter. We appreciate the opportunity to clarify this matter.

**Journal Requirement** **5**. Thank you for stating the following financial disclosure: 
“This research was financially supported by a grant from the Faculty of Medicine, Prince of Songkla University, Thailand.”
We note that one or more of the authors is affiliated with the funding organization, indicating the funder may have had some role in the design, data collection, analysis or preparation of your manuscript for publication; in other words, the funder played an indirect role through the participation of the co-authors. If the funding organization did not play a role in the study design, data collection and analysis, decision to publish, or preparation of the manuscript and only provided financial support in the form of authors' salaries and/or research materials, please do the following:
1. Review your statements relating to the author contributions, and ensure you have specifically and accurately indicated the role(s) that these authors had in your study. These amendments should be made in the online form.
2. Confirm in your cover letter that you agree with the following statement, and we will change the online submission form on your behalf: 
“The funder provided support in the form of salaries for authors [insert relevant initials], but did not have any additional role in the study design, data collection and analysis, decision to publish, or preparation of the manuscript. The specific roles of these authors are articulated in the ‘author contributions’ section.

### ****Response:****

We thank the Editor for this important clarification regarding the role of the funding organization.

We confirm that the Faculty of Medicine, Prince of Songkla University, Thailand, did not have any role in the study design, data collection and analysis, decision to publish, or preparation of the manuscript.

The funding organization provided research grant support for the conduct of the study. All authors are employees of Prince of Songkla University and receive institutional salaries as part of their academic appointments; however, the institution had no additional role in the scientific conduct, analysis, interpretation, or reporting of the study. All scientific and publication-related decisions were made independently by the authors.

We agree with and confirm the following statement:

**“The funder provided support in the form of salaries for authors [PL, MT], but did not have any additional role in the study design, data collection and analysis, decision to publish, or preparation of the manuscript. The specific roles of these authors are articulated in the ‘Author Contributions’ section.”**

We confirm that the Author Contributions section has been carefully reviewed to ensure that each author’s role is clearly and accurately described in the online submission system.

**Response to Reviewer #1 comments**

**Comment 1:** While vitamin C has shown potential in reducing chemotherapy-induced toxicities in other cancers – Sentence changed to chemotherapy-induced toxicities in some of cancers.

### ****Response:****

We sincerely thank the Reviewer for this helpful suggestion. We agree that the original wording may have implied broader generalizability than supported by the available evidence. To improve precision and avoid overstatement, we have revised the sentence accordingly.

**The revised Abstract (page 2, paragraph 1, lines 26-27)**

“While vitamin C has shown potential in reducing chemotherapy-induced toxicities in some cancers”

**Comment 2:** Common adverse effects include gastrointestinal symptoms, such as nausea, anorexia, oral mucositis, diarrhea, and dysphagia, hematological toxicities (e.g., neutropenia, anemia, and thrombocytopenia), electrolyte imbalances (e.g., hyponatremia and hypokalemia). – Reference should be include.

### ****Response:****

We sincerely thank the Reviewer for this helpful suggestion. We agree that the statement describing common adverse effects of chemoradiotherapy should be appropriately supported by references. Accordingly, we have added relevant citations in the Introduction to substantiate this statement.

**The revised Introduction** (page 3, paragraph 1, lines 62–65)

Common adverse effects include gastrointestinal symptoms, such as nausea, anorexia, oral mucositis, diarrhea, and dysphagia, hematological toxicities (e.g., neutropenia, anemia, and thrombocytopenia), and electrolyte imbalances (e.g., hyponatremia and hypokalemia) [7,9].

**Comment 3:** Recent studies have largely focused on IVC, with doses ranging from 1 to 10 g per session – How many sessions (per day or something else?) It needs to be clarified in the manuscript.

### ****Response:****

### We agree that clarification regarding the dosing schedule of intravenous vitamin C (IVC) in prior studies was necessary. Accordingly, we have revised the Introduction to specify that the reported dose range (1–10 g) refers to the amount administered per infusion, and that the frequency of administration varies across studies.

**The revised Introduction** (page 5, paragraph 1, lines 95–100):

Recent studies have largely focused on IVC, with doses ranging from 1 to 10 g per session. The frequency of administration ranges from once per chemotherapy cycle to several infusions per week, depending on study design and clinical protocol. These regimens have shown potential benefits in reducing chemotherapy-related toxicities and improving quality of life in patients with ovarian, colorectal, and hematological malignancies [10,11].

**Comment 4:** Sixty-eight patients diagnosed with NPC and scheduled for CCRT were enrolled. – Which stage of NPC patients were chosen for this study?

### ****Response:****

We sincerely thank the Reviewer for this valuable comment. We agree that the clinical stage of enrolled patients should be clearly specified in the Methods section.

In this study, we included patients with stage II–IVa nasopharyngeal carcinoma who were eligible for concurrent chemoradiotherapy (CCRT), consistent with standard treatment indications. Staging was determined according to the American Joint Committee on Cancer (AJCC) Cancer Staging Manual, 8th edition.

**The revised Methods – Study Participants section** (page 6, paragraph 2, lines 131–133):

The inclusion criteria required participants to be aged 18 years or older, have a confirmed NPC diagnosis, clinical stage II–IVa according to the **American Joint Committee on Cancer** (AJCC) Cancer Staging Manual, 8th edition, and be eligible for CCRT.

**Comment 5:** Regarding laboratory parameters, reductions in platelet count and serum sodium level were significantly less in the vitamin C group compared with the placebo group. – As per your results in the tables, there’s no difference in vitamin C and placebo groups.

### ****Response:****

### We sincerely thank the Reviewer for this careful observation. We agree that clarification was necessary to distinguish between baseline comparisons and longitudinal treatment effects.

### As shown in Table 1, baseline laboratory values were similar between the vitamin C and placebo groups. The statistically significant findings for platelet count and serum sodium levels were identified in the longitudinal analysis evaluating changes over time between groups, based on linear mixed-effects modeling. Specifically, these differences reflect significant group-by-time interaction effects rather than cross-sectional baseline comparisons.

### To improve clarity and prevent misinterpretation, we have revised the Results section accordingly.

**The revised Results** (page 14, paragraph 1, lines 257-266):

**Hematologic and biochemical parameters**

In longitudinal mixed-effects analyses, significant group-by-time interaction effects were observed for platelet counts (p = 0.023) and serum sodium levels (p = 0.021), indicating differential changes over time between groups. Fig 2 displays the median and interquartile ranges (IQRs) before and after chemotherapy. The median platelet count decreased from 313 ×10³ cells/µL to 276 ×10³ cells/µL in the vitamin C group, compared with a decrease from 306 ×10³ cells/µL to 220 ×10³ cells/µL in the placebo group. Similarly, median serum sodium levels remained relatively stable in the vitamin C group (136.8 to 137.9 mmol/L), whereas a decline was observed in the placebo group (138.8 to 134.2 mmol/L).

**Comment 6:** However, platelet count reductions were significantly less in the vitamin C group than in the placebo group following chemotherapy – Mention the values in the manuscript.

### ****Response:****

We sincerely thank the Reviewer for this valuable suggestion. We agree that reporting the numerical values would improve clarity and allow readers to better appreciate the magnitude of the observed differences.

Accordingly, we have revised the Results section to include the median values for platelet counts and serum sodium levels before and after chemotherapy.

**The revised Results** (page 14, paragraph 1, lines 261-266):

The median platelet count decreased from 313 ×10³ cells/µL to 276 ×10³ cells/µL in the vitamin C group, compared with a decrease from 306 ×10³ cells/µL to 220 ×10³ cells/µL in the placebo group. Similarly, median serum sodium levels remained relatively stable in the vitamin C group (136.8 to 137.9 mmol/L), whereas a decline was observed in the placebo group (138.8 to 134.2 mmol/L).

**Comment 7:** Abbreviations should be included in the manuscript.

### ****Response:****

### We sincerely thank the Reviewer for this helpful comment. We agree that all abbreviations should be clearly defined at their first occurrence to improve clarity and readability. Accordingly, we have carefully reviewed the manuscript and ensured that all abbreviations are defined upon first use and used consistently throughout the text.

### The revised manuscript now includes appropriate definitions for abbreviations such as intravenous vitamin C (IVC), concurrent chemoradiotherapy (CCRT), chemotherapy (CMT), **American Joint Committee on Cancer** (AJCC), Common Terminology Criteria for Adverse Events (CTCAE), white blood cell (WBC), and interquartile range (IQR).

**Comment 8:** Quality of figures must be improved.

### ****Response:****

We sincerely thank the Reviewer for this helpful comment. We agree that improving figure quality enhances the clarity and overall presentation of the manuscript.

Accordingly, Fig 1 and 2 have been regenerated and uploaded in high-resolution TIFF format (600 dpi) in accordance with the journal’s figure preparation guidelines. Font size, alignment, and labeling have also been optimized to improve readability.

The revised high-resolution figures have been submitted with the updated manuscript.

**The revised Fig 1 and Fig 2** have been uploaded as “revised Fig_1.tiff.” and “revised Fig_2.tiff.”

**Comment 9:** Based on your results there’s not much improvement of adverse effects from chemotherapy. What is your conclusion about your study?

### ****Response:****

### We sincerely thank the Reviewer for this thoughtful comment. We agree that our study did not demonstrate a statistically significant reduction in gastrointestinal adverse effects associated with chemotherapy, and the overall impact of intravenous vitamin C (IVC) on most clinical toxicities was modest.

### However, longitudinal analysis demonstrated a significant attenuation of platelet count decline in the IVC group compared with placebo. As platelet count was a secondary outcome, we have carefully revised the Conclusion section to ensure a balanced and appropriately cautious interpretation of this finding.

**The revised Conclusion** (page 19, paragraph 2, lines 378-384):

IVC administration did not significantly reduce gastrointestinal adverse events in patients with nasopharyngeal carcinoma undergoing CCRT. However, it was associated with attenuation of platelet count decline during treatment. As this was a secondary outcome, preservation of platelet levels may help maintain treatment intensity and reduce the risk of bleeding or chemotherapy interruptions. This observation suggests a potential supportive effect of IVC on platelet-related toxicity during CCRT, although confirmatory studies are required.

**Comment 10:** Why are you choosing 2 g IVC for your study? As per literature it was 1–10 g, please explain these in the manuscript.

### ****Response:****

### We sincerely thank the Reviewer for this important comment. We agree that further clarification regarding the rationale for selecting the 2 g intravenous vitamin C (IVC) dose is necessary.

The 2 g dose was selected based on safety considerations and integration with routine clinical practice. Intravenous vitamin C is recommended to be administered as a slow infusion at approximately 33 mg per minute to ensure tolerability. At our institution, patients undergo a standardized 1-hour pre-chemotherapy hydration period prior to cisplatin administration. At the recommended infusion rate, this timeframe permits delivery of approximately 2 g of vitamin C (33 mg/min × 60 minutes ≈ 2,000 mg) without extending treatment time or disrupting the standard chemotherapy workflow. Therefore, 2 g represents the maximal dose that can be safely administered within the existing prehydration protocol.

To clarify this rationale, we have revised the Discussion section accordingly.

**The revised Discussion** (page 17, paragraph 2, lines 338-347):

In this study, a 2 g dose of IVC administered over 1 hour prior to chemotherapy was selected in accordance with our institution’s standardized pre-chemotherapy hydration protocol. IVC is administered as a slow infusion (approximately 33 mg/min) to ensure safety and tolerability [29]. Given the routine 1-hour prehydration period before cisplatin administration, this timeframe permits delivery of up to 2 g without altering standard clinical workflow. This dosing strategy was chosen to evaluate the feasibility and potential supportive effects of IVC within routine clinical practice. This approach is further supported by pharmacokinetic evidence demonstrating that intravenous administration produces markedly higher plasma ascorbate concentrations than oral supplementation and achieves predictable systemic exposure [16].

**Response to Reviewer #2 comments**

## **Comment 1:** Introduction needs enhancement

**Response:**

We sincerely thank the Reviewer for this important comment. In response, we have substantially revised and strengthened the Introduction to enhance clarity, scientific rationale, and contextual relevance. Specifically, we expanded the clinical context of chemotherapy-related toxicities in NPC, incorporated additional literature regarding the potential supportive role of intravenous vitamin C (IVC)—particularly in relation to hematologic effects—clarified the pharmacokinetic differences between oral and intravenous administration, and more clearly articulated the knowledge gap and study objective. The revised Introduction has been updated accordingly and is highlighted in the revised manuscript.

The revised Introduction (pages 3–5, lines 45–113):

Introduction

Nasopharyngeal carcinoma (NPC) is an epithelial malignancy arising in the nasopharynx, a unique subsite of the head and neck region. NPC is geographically distributed in a distinct manner, with the highest incidence observed in East and Southeast Asia, including Southern China, Malaysia, Vietnam, and Thailand [1]. In these regions, NPC is a major public health concern, especially among men, with incidence rates reaching up to 10–20 cases per 100,000 individuals in endemic areas [2,3]. In Thailand, NPC accounts for approximately 1–3% of all cancers but is a leading cause of cancer-related morbidity in men aged 30 to 60 years [2]. NPC presents a significant clinical challenge owing to its deep anatomical location and nonspecific early symptoms—such as nasal congestion, epistaxis, headache, and hearing issues—which frequently lead to delayed diagnosis. Consequently, over 70% of cases are detected at advanced stages (stages III–IV), where prognosis is substantially worse than that in early-stage disease. While early-stage NPC has a 5-year overall survival (OS) exceeding 80%, advanced-stage disease may reduce OS to below 60% [4,5]. The primary treatment for NPC is concurrent chemoradiotherapy (CCRT), which combines radiotherapy with platinum-based chemotherapy (CMT) [6]. While CCRT has demonstrated efficacy in improving survival outcomes, it is often associated with various toxicities [7,8]. Common adverse effects include gastrointestinal symptoms, such as nausea, anorexia, oral mucositis, diarrhea, and dysphagia, hematological toxicities (e.g., neutropenia, anemia, and thrombocytopenia), and electrolyte imbalances (e.g., hyponatremia and hypokalemia) [7,9]. These adverse effects may necessitate dose reductions or lead to treatment delays and interruptions, thereby potentially compromising therapeutic effectiveness and overall survival.

Vitamin C (ascorbic acid) has garnered attention as a potential supportive therapy in oncology due to its antioxidant and anti-inflammatory properties. Since the 1970s, vitamin C has been investigated as a supportive treatment in oncology, with accumulating evidence suggesting its ability to alleviate chemotherapy-related symptoms such as nausea, vomiting, fatigue, insomnia, appetite loss, and cancer-related pain [10]. Moreover, intravenous vitamin C (IVC) has been explored for its potential role in mitigating chemotherapy-induced hematologic toxicities, particularly thrombocytopenia, through reduction of oxidative stress within the bone marrow microenvironment and preservation of megakaryocyte function [11,12]. For example, studies in ovarian cancer have reported reduced bone marrow toxicity and improved platelet recovery with adjunctive IVC administration [11], while investigations in hematologic malignancies have suggested potential restoration of hematopoietic function, including platelet counts [12]. However, findings across cancer types remain heterogeneous, and evidence in head and neck cancers, including nasopharyngeal carcinoma, is limited. The rationale for considering vitamin C as a supportive intervention in oncology is grounded in its antioxidant and immunomodulatory properties. Chemotherapy-related toxicities are, in part, driven by increased oxidative burden and activation of inflammatory pathways, which contribute to mucosal damage and suppression of hematopoiesis. By attenuating oxidative stress and regulating inflammatory signaling, IVC may help mitigate treatment-related cellular injury and support the maintenance of hematologic stability during cytotoxic therapy [13,14]. Pharmacokinetic studies have demonstrated that intravenous administration produces markedly higher plasma ascorbate concentrations than oral supplementation due to limitations in intestinal absorption and renal clearance. Padayatty et al. further clarified these differences, showing that oral administration produces only moderate plasma concentrations, whereas intravenous infusion results in rapid and markedly elevated levels [15,16]. These pharmacokinetic properties provide a rationale for the use of IVC rather than oral supplementation in the context of chemotherapy-related toxicity management. Recent studies have largely focused on IVC, with doses ranging from 1 to 10 g per session. The frequency of administration ranges from once per chemotherapy cycle to several infusions per week, depending on study design and clinical protocol. These regimens have shown potential benefits in reducing chemotherapy-related toxicities and improving quality of life in patients with ovarian, colorectal, and hematological malignancies [10,11]. Safe IVC administration has been well documented when delivered by slow infusion under appropriate monitoring, with no harm to normal tissues, further supporting its application in oncology [11,17].

Despite growing evidence supporting the role of IVC in oncology supportive care, its specific effects in patients with NPC undergoing CCRT remain largely unexplored. Particularly, the impact of IVC on gastrointestinal toxicities during CCRT has not been well characterized in this population. Furthermore, its potential effects on hematologic parameters—including platelet preservation—and electrolyte abnormalities during cisplatin-based therapy have not been comprehensively elucidated [10]. Notably, randomized controlled trials evaluating IVC in this specific clinical setting remain scarce. Therefore, we conducted a prospective, randomized, double-blind, placebo-controlled trial to evaluate whether IVC reduces chemotherapy-induced gastrointestinal adverse events in patients with nasopharyngeal carcinoma undergoing CCRT, with secondary exploratory analyses of hematologic and electrolyte outcomes.

### Comment 2: Author missed the rational study objection.

### ****Response:****

### We sincerely thank the Reviewer for this important comment. We agree that the study rationale and objective should be clearly and explicitly articulated.

### In response, we have revised the final paragraph of the Introduction to clearly define the clinical problem, the existing knowledge gap, and the primary study objective. Specifically, we clarified that the primary objective was to evaluate whether intravenous vitamin C (IVC) reduces chemotherapy-induced gastrointestinal adverse events in patients with nasopharyngeal carcinoma undergoing concurrent chemoradiotherapy (CCRT), with secondary exploratory evaluation of hematologic and electrolyte outcomes.

The revised Introduction (page 5, paragraph 2, lines 110–113):

Therefore, we conducted a prospective, randomized, double-blind, placebo-controlled trial to evaluate whether IVC reduces chemotherapy-induced gastrointestinal adverse events in patients with nasopharyngeal carcinoma undergoing CCRT, with secondary exploratory analyses of hematologic and electrolyte outcomes.

**Comment 3: Author provide the FDA toxicological data**

### ****Response:****

### We sincerely thank the Reviewer for this important comment. We agree that clarification regarding regulatory approval and safety of the intravenous vitamin C preparation is warranted.

### The vitamin C injection used in this study is a pharmaceutical product approved by the Thai Food and Drug Administration (Thai FDA registration number: 1A 1557/30). The formulation has undergone regulatory evaluation for quality, safety, and toxicological compliance in accordance with national pharmaceutical standards. The dose administered in this study (2 g infused over 1 hour) falls within approved clinical use parameters and is routinely used in standard medical practice.

### To address this concern, we have added clarification regarding regulatory approval and product safety in the Methods section.

**The revised Methods section** (page 7, paragraph 1, lines 147–150):

The IVC formulation used in this study is an approved pharmaceutical product registered with the Thai Food and Drug Administration (registration number: 1A 1557/30). The preparation complies with national regulatory standards and was administered in accordance with standard institutional safety monitoring procedures.

**Comment 4: Methodological part need to improve.**

**Response:**

We sincerely thank the Reviewer for this constructive comment. In response, we have carefully revised and expanded the Methods section to enhance clarity, methodological transparency, and reproducibility.

Specifically, we clarified the study design and trial registration details; expanded the description of randomization, allocation concealment, and blinding procedures; refined the intervention protocol including dosing, preparation, and regulatory approval; clearly defined the primary and secondary outcomes; and strengthened the Statistical Analysis section by detailing the analytical framework, including linear mixed-effects modeling and complementary Bayesian analysis. We believe these revisions substantially improve the methodological rigor and reporting transparency of the study.

The revised Materials and methods section (page 5-10, lines 115–214):

Materials and methods

**Study design**

This prospective, randomized, double-blind, placebo-controlled trial was conducted at Prince of Songkla University, Thailand, between March 14, 2019 and December 20, 2020. The study protocol was approved by the Ethics Committee of the Faculty of Medicine, Prince of Songkla University (REC.61-416-13-1) and registered with the Thai Clinical Trial Registry (TCTR20190316003) on March 16, 2019. The study was submitted to the Thai Clinical Trial Registry at study initiation; however, due to administrative review and clarification requests, the official registration date appears after enrollment began. Ethics approval and the finalized protocol were completed prior to participant enrollment. The authors confirm that all ongoing and related trials for this intervention are registered. The study protocol adhered to relevant guidelines and regulations, including the World Medical Association Declaration of Helsinki on ethical principles for medical research involving human participants.

**Study participants**

Sixty-eight patients diagnosed with NPC and scheduled for CCRT were enrolled. The inclusion criteria required participants to be aged 18 years or older, have a confirmed NPC diagnosis, clinical stage II–IVa according to the **American Joint Committee on Cancer** (AJCC) Cancer Staging Manual, 8th edition, and be eligible for CCRT. The exclusion criteria included a history of hypersensitivity to vitamin C, contraindications to chemotherapy, glucose-6-phosphate dehydrogenase (G-6-PD) deficiency (confirmed through laboratory testing), a history of kidney stones, and previous radiation therapy. Participant flow was monitored throughout the study. Reasons for loss to follow-up were documented, including one withdrawal and one transferred to a different hospital in the Vitamin C group, and one treatment refusal in the placebo group. All remaining participants completed the planned course of chemoradiotherapy and intervention.

**Randomization and intervention**

All participants provided written informed consent before enrollment. Randomization was performed using a computer-generated random sequence with a 1:1 allocation ratio. Allocation concealment was ensured using sequentially numbered, opaque, sealed envelopes prepared by an independent investigator not involved in patient recruitment or outcome assessment. Group A received 2 g of IVC 1 h before chemotherapy. The IVC formulation used in this study is an approved pharmaceutical product registered with the Thai Food and Drug Administration (registration number: 1A 1557/30). The preparation complies with national regulatory standards and was administered in accordance with standard institutional safety monitoring procedures. The preparation involved removing 8 mL of 5% dextrose in water (DW) and replacing it with 2 g (8 mL) of vitamin C, resulting in a final volume of 50 mL, which was infused over 1 h. Group B received a placebo consisting of 50 mL of 5% DW without vitamin C administered over the same duration. The vitamin C and placebo solutions were identical in appearance, color, and volume. To ensure blinding, chemotherapy nurses not involved in the study prepared the solutions. The study was double-blinded, with participants, treating physicians, outcome assessors, and data analysts unaware of group allocation.

All patients underwent CCRT, consisting of platinum-based chemotherapy and external beam radiation. The chemotherapy regimen included three cycles of intravenous cisplatin (100 mg/m²) administered every three weeks. Vitamin C or placebo was administered only on the days that cisplatin was delivered. If chemotherapy was delayed, the corresponding dose of vitamin C or placebo was also withheld. All patients who completed chemotherapy received all three planned doses of the assigned intervention. Radiotherapy was delivered using intensity-modulated radiation therapy, with a total dose of 70 Gy in 35 fractions over seven weeks. The radiotherapy protocol was standardized and consistent across both treatment groups.

**Data collection**

Data collected included patient demographics (sex, age, and weight), cancer histologic type, tumor differentiation, and cancer stage (following the AJCC Cancer Staging Manual, 8th edition). Information on chemotherapy dates and cycles was also recorded. Adverse effects were evaluated both prior to each chemotherapy cycle and at nadir time points (days 7–14 post-infusion), in accordance with the institutional protocol, using the Common Terminology Criteria for Adverse Events (CTCAE) version 5.0. Physical examinations and laboratory tests were conducted to monitor adverse effects. The collected parameters included gastrointestinal symptoms such as nausea, anorexia, oral mucositis, diarrhea, and dysphagia, white blood cell (WBC) counts, hemoglobin levels, platelet counts, creatinine levels, serum sodium and potassium levels. Adverse events were classified from Grade 1 (mild) to Grade 5 (death) according to CTCAE version 5.0 [18]. The primary outcome was the incidence of gastrointestinal adverse events graded according to CTCAE version 5.0 after the chemotherapy cycles. Hematologic (white blood cell count, hemoglobin level, and platelet count) and biochemical parameters (serum creatinine, sodium, and potassium levels) were classified as exploratory secondary outcomes.

**Statistical analysis**

All analyses were performed according to the per-protocol approach using R software (R Foundation for Statistical Computing, Vienna, Austria). An intention-to-treat analysis was not performed due to the minimal loss to follow-up and complete outcome data among randomized participants. Descriptive statistics were summarized as frequencies, means with standard deviations (SDs), or medians with interquartile ranges (IQRs), as appropriate. Between-group comparisons for categorical variables were performed using the chi-square test or Fisher’s exact test, as appropriate. Continuous variables were analyzed using independent *t*-tests or nonparametric equivalents when normality assumptions were not met. Longitudinal laboratory parameters were analyzed using linear mixed-effects models with random intercepts to account for within-subject correlations across repeated measurements. Maximum likelihood estimation was used for parameter estimation. For graphical presentation, observed median values with IQRs were displayed without baseline normalization or delta transformation. Inter-patient variability was addressed through the mixed-effects modeling framework.

The sample size was calculated to detect a reduction in the incidence of gastrointestinal adverse events. The calculation was based on the proportions reported in a previous study by Ma et al. [11], in which gastrointestinal adverse events occurred in 9 of 13 patients (69%) in the vitamin C group and in 12 of 12 patients (100%) in the control group, corresponding to an anticipated absolute risk reduction of approximately 31%. Using these proportions, with 80% power and a two-sided alpha of 0.05, the required sample size was estimated to be 22 participants per group. To account for potential attrition, additional participants were enrolled. In addition to frequentist analyses, Bayesian logistic regression models were fitted using weakly informative priors to estimate posterior probability distributions of treatment effects. This approach allows direct probabilistic interpretation of the direction and magnitude of treatment effects, which is particularly useful in the context of exploratory secondary outcomes. Results are summarized using posterior probabilities of benefit or harm. Treatment effects were interpreted according to whether the posterior distribution indicated a high probability of benefit or harm. The Bayesian analysis was included as a complementary exploratory framework to provide additional inferential insight.

**Comment 5: Author need to rewrite result part with statistical comparison.**

**Response:**

We sincerely thank the Reviewer for this valuable suggestion. In response, we have comprehensively revised and reorganized the Results section to improve clarity, structure, and presentation of statistical comparisons.

Specifically, we have:

1. Clearly presented the primary endpoint—the incidence of Grade 1–2 gastrointestinal adverse events during three chemotherapy cycles—and explicitly reported the corresponding p-values for each symptom category (nausea, anorexia, oral mucositis, diarrhea, and dysphagia). We have clearly stated that no statistically significant differences were observed between the vitamin C and placebo groups.
2. Clarified that hematologic and biochemical parameters reported statistically significant between-group differences for platelet counts (p = 0.023) and serum sodium levels (p = 0.021).

**Revised Results**

**Gastrointestinal adverse events** (page 12, paragraph 2**,** lines 244-250)

The incidence of Grade 1–2 gastrointestinal adverse events did not differ significantly between the vitamin C and placebo groups. Specifically, no significant between-group differences were observed for nausea (p = 0.23), anorexia (p = 0.89), oral mucositis (p = 1.00), diarrhea (p = 0.24), or dysphagia (p = 0.24). Notably, only a small number of patients experienced Grade 3 events, and no Grade 4 toxicities were observed in our study (Table 2).

**Hematologic and biochemical parameters** (page 14, paragraph 1**,** lines 257-266)

In longitudinal mixed-effects analyses, significant group-by-time interaction effects were observed for platelet counts (p = 0.023) and serum sodium levels (p = 0.021), indicating differential changes over time between groups. Fig 2 displays the median and interquartile ranges (IQRs) before and after chemotherapy. The median platelet count decreased from 313 ×10³ cells/µL to 276 ×10³ cells/µL in the vitamin C group, compared with a decrease from 306 ×10³ cells/µL to 220 ×10³ cells/µL in the placebo group. Similarly, median serum sodium levels remained relatively stable in the vitamin C group (136.8 to 137.9 mmol/L), whereas a decline was observed in the placebo group (138.8 to 134.2 mmol/L).

**Comment 6:** Discussion is Very short, author need rewrite mechanistic discussion with result comparison
**Response:**

We sincerely thank the Reviewer for this valuable comment. In response, we have substantially expanded and refined the Discussion section to provide a more comprehensive interpretation of the findings.

Specifically, we have:

1. Elaborated on addressing the absence of significant reduction in gastrointestinal toxicities and discussing potential explanations for this finding.
2. Expanded the discussion of secondary findings, particularly the observed attenuation of platelet decline in the vitamin C group, and provided a detailed biological rationale supported by relevant literature.
3. Discussed the mechanistic basis of vitamin C in reducing oxidative stress, preserving megakaryocyte function, and maintaining bone marrow microenvironment stability.
4. Included discussion of contrasting findings from prior studies to provide balanced interpretation.

**Revised Discussion** (page 14-19**,** lines 275-375):

Discussion

Chemotherapy-induced side effects can substantially compromise treatment efficacy and diminish overall survival in patients with nasopharyngeal carcinoma. In this randomized, double-blind, placebo-controlled trial, IVC administered prior to cisplatin-based chemoradiotherapy did not significantly reduce the incidence of Grade 1–2 gastrointestinal adverse events, nor did it significantly affect other hematologic parameters (e.g., white blood cell count and hemoglobin level) or metabolic indices. However, longitudinal analysis demonstrated a significantly attenuated decline in platelet counts in the vitamin C group compared with placebo.

Previous studies investigating the effects of vitamin C on chemotherapy-related gastrointestinal toxicities have reported similar findings; the absence of significant differences in our study aligns with these earlier observations. For example, Wang et al. observed no reduction in gastrointestinal adverse events in patients with colorectal cancer treated with high-dose vitamin C alongside FOLFOX-based chemotherapy [19], while Pathak et al. found no clinical benefit of high-dose antioxidants, including vitamin C, in patients with lung cancer receiving chemotherapy [20]. Similarly, Zasowska-Nowak et al. reported insufficient data to draw definitive conclusions regarding the effectiveness of IVC therapy in reducing chemotherapy-induced toxicity in patients with advanced-stage cancer [21]. The lack of observed effects in our study may be explained by several factors. First, most adverse events were of low grade (Grade 1), which may have limited the observable benefit of any supportive intervention. Second, gastrointestinal symptoms during chemoradiotherapy result from multifactorial mechanisms, including mucosal inflammation, microbiome disruption, and cytokine dysregulation, which may not be fully mitigated by the antioxidant or anti-inflammatory actions of vitamin C alone [22–24].

Despite the modest clinical differences, our study found a smaller reduction in platelet counts during CCRT in patients with NPC who received IVC than in those in the placebo group. This observation aligns with previous research findings suggesting that vitamin C may help mitigate chemotherapy-induced hematological toxicities. Foster et al. studied the effects of IVC in patients with acute myeloid leukemia undergoing chemotherapy and found improvements in hematological indices, including platelet counts, suggesting that vitamin C may help restore normal hematologic function [12]. Similarly, Monti et al. conducted a clinical trial involving patients with pancreatic cancer undergoing chemotherapy and found that most patients who received IVC experienced only Grade 1 thrombocytopenia. They proposed that ascorbate may not disturb rapidly dividing normal cells, such as those within the bone marrow [25]. Furthermore, Ma et al. studied patients with ovarian cancer undergoing chemotherapy and found that those who received IVC had significantly lower bone marrow toxicities than those who did not [11]. Their findings suggest that vitamin C may protect hematopoietic progenitor cells from chemotherapy-induced damage, preserve platelet production, and limit thrombocytopenia. Although our results and those of other studies indicate a protective effect of IVC on platelet counts, some studies have reported contrasting results. For example, Wang et al. found no significant difference in thrombocytopenia between vitamin C and control groups in patients with colorectal cancer undergoing chemotherapy [19]. Similarly, Pathak et al. observed no significant impact of IVC on chemotherapy toxicity in patients with lung cancer [20]. These inconsistencies may stem from differences in baseline nutritional status, cancer type, and chemotherapy regimen. In our study, the concurrent use of vitamin C and chemotherapy may have enhanced its protective effects. The mechanism through which vitamin C helps prevent platelet count reduction probably involves reducing oxidative stress and maintaining bone marrow integrity. Chemotherapy-induced oxidative damage is believed to harm megakaryocytes, the precursor cells responsible for platelet production. Vitamin C may neutralize reactive oxygen species (ROS), reduce oxidative stress, preserve megakaryocyte function, and potentially limit excessive platelet depletion [26]. By scavenging ROS, vitamin C mitigates oxidative damage in the bone marrow microenvironment, which is crucial for maintaining the health and function of hematopoietic stem cells and megakaryocytes. Additionally, vitamin C supports endothelial cell function and promotes collagen synthesis, vital for maintaining vascular health and stability. Collagen is crucial for platelet adhesion and aggregation. Therefore, adequate vitamin C levels help maintain the structural integrity of blood vessels, reducing platelet activation and destruction during chemotherapy [27]. Moreover, vitamin C may attenuate the inflammatory responses [28] that contribute to chemotherapy-induced thrombocytopenia, further supporting platelet homeostasis.

In this study, a 2 g dose of IVC administered over 1 hour prior to chemotherapy was selected in accordance with our institution’s standardized pre-chemotherapy hydration protocol. IVC is administered as a slow infusion (approximately 33 mg/min) to ensure safety and tolerability [29]. Given the routine 1-hour prehydration period before cisplatin administration, this timeframe permits delivery of up to 2 g without altering standard clinical workflow. This dosing strategy was chosen to evaluate the feasibility and potential supportive effects of IVC within routine clinical practice. This approach is further supported by pharmacokinetic evidence demonstrating that intravenous administration produces markedly higher plasma ascorbate concentrations than oral supplementation and achieves predictable systemic exposure [16]. Additionally, this dosing strategy has been demonstrated to be safe and well tolerated in patients with advanced malignancies, with no reported infusion-related adverse events [25,30]. While significant differences were observed in platelet counts between the IVC and control groups, these values largely remained within normal or near-normal clinical ranges. No overt bleeding episodes were reported during the study period, suggesting limited immediate clinical relevance. However, maintaining more stable platelet counts during chemoradiotherapy may help reduce the risk of bleeding, minimize the need for platelet transfusions, and prevent treatment delays or dose reductions. Such benefits could translate into better tolerance of chemoradiotherapy, improved treatment adherence, fewer unplanned hospital visits, and overall improvements in patients’ clinical outcomes. Nevertheless, owing to the exploratory nature of the findings and the limited clinical outcome data, further validation in larger, prospective studies is warranted.

Our study had some limitations. The small sample size may have limited the statistical power to detect subtle or moderate effects of vitamin C on various adverse outcomes, particularly gastrointestinal symptoms and metabolic parameters. Furthermore, the short-term follow-up—limited to three chemotherapy cycles—may not have captured the cumulative effects of treatment. Longer follow-up periods are needed to assess potential long-term benefits or risks. The 2 g dose of vitamin C used may have been insufficient to elicit more pronounced therapeutic effects. Higher doses or more frequent administration might result in greater reductions in other chemotherapy-related side effects. Future studies should address the limitations of the present study by enrolling larger participant groups and extending the follow-up period to evaluate the sustained effects of vitamin C on toxicities. Conducting multicenter randomized controlled trials would enhance the generalizability of the findings and provide greater statistical power. Additionally, investigating alternative dosing regimens of IVC, as well as its potential combined effect with other antioxidants or supportive care agents, may further optimize its therapeutic impact. **Collectively, these efforts will help clarify the clinical utility of IVC as an adjunctive therapy in nasopharyngeal carcinoma.**

**Comment 7:** Conclusion not covey the correct way

**Response:**
We sincerely thank the Reviewer for this valuable comment. We agree that the Conclusion should clearly and accurately reflect the primary findings of the study while appropriately contextualizing the observed secondary outcomes. In response, we have revised the Conclusion to explicitly state the absence of a significant reduction in gastrointestinal adverse events and to clarify that the attenuation of platelet count decline was observed as a secondary outcome. We have also emphasized the exploratory nature of this finding and the need for confirmatory studies to avoid overinterpretation.

**The revised Conclusion** (page 19, paragraph 2, lines 377-384):

**Conclusions**

IVC administration did not significantly reduce gastrointestinal adverse events in patients with nasopharyngeal carcinoma undergoing CCRT. However, it was associated with attenuation of platelet count decline during treatment. As this was a secondary outcome, preservation of platelet levels may help maintain treatment intensity and reduce the risk of bleeding or chemotherapy interruptions. This observation suggests a potential supportive effect of IVC on platelet-related toxicity during CCRT, although confirmatory studies are required.

**Comment 8: Figure 2 very poor presentation and all the statistical error look like similar, author recheck the statistical error value.**

**Response:**

We sincerely thank the Reviewer for this important comment. We agree that clear graphical presentation and accurate representation of statistical variability are essential for proper interpretation of the results.

In response, we carefully re-examined all statistical computations underlying Figure 2, including the linear mixed-effects model outputs, descriptive summary statistics, and graphical plotting procedures. Independent re-verification of the numerical outputs confirmed that the reported values and associated p-values are accurate and fully consistent with the statistical methods described in the manuscript.

We note that the error bars in Figure 2 represent observed interquartile ranges (IQRs) rather than model-based standard errors. The apparent similarity in some error bars reflects comparable variability across time points and groups, rather than duplication or computational error.

To improve clarity and presentation quality, we have revised Figure 2 as follows:
• Enhanced image resolution (600 DPI)
• Explicitly labeled error bars as IQR
• Optimized axis scaling to better distinguish between-group trajectories
• Standardized formatting and spacing to improve visualization of dispersion

**The revised Figure 2** has been uploaded as “revised Fig_2.tiff.”

**Response to Reviewer #3 comments**

**Comment 1: The abstract contains too much methodological detail. It should be revised to briefly describe the study design and focus on the key findings.**

**Response:**
We sincerely thank the Reviewer for this valuable comment. We agree that the Abstract should concisely describe the study design while emphasizing the principal findings and their clinical relevance.

In response, we have revised the Abstract to shorten methodological details and focus more clearly on the key outcomes. Specifically, we reduced procedural descriptions and retained only essential design elements (randomized, double-blind, placebo-controlled trial), while highlighting the absence of significant differences in gastrointestinal adverse events and the observed attenuation of platelet count decline in the intravenous vitamin C group.

**The revised Abstract** (page 2, paragraph 1, lines 22–37):

Nasopharyngeal carcinoma is prevalent in Thailand, with a substantial proportion of cases diagnosed at advanced stages. The standard treatment, concurrent chemoradiotherapy, is associated with considerable adverse effects, which may compromise therapeutic efficacy and diminish patients' quality of life. While vitamin C has shown potential in reducing chemotherapy-induced toxicities in some cancers, its effects in nasopharyngeal carcinoma remain unclear. In this randomized, double-blind, placebo-controlled trial, patients with nasopharyngeal carcinoma undergoing concurrent chemoradiotherapy were assigned to receive either 2 g of intravenous vitamin C or placebo prior to chemotherapy. The incidence of gastrointestinal adverse effects—including nausea, anorexia, mucositis, diarrhea, and dysphagia—did not differ significantly between groups. However, longitudinal analysis demonstrated a significantly attenuated decline in platelet counts in the vitamin C group compared with placebo. Although intravenous vitamin C did not reduce gastrointestinal toxicities, the observed platelet preservation suggests a potential supportive effect that warrants further investigation.

**Comment 2: The manuscript lacks a detailed description of how hematological values were adjusted for individual patients in the graphical representations. Please clarify whether baseline normalization, delta changes, or other statistical adjustments were applied to account for inter-patient variability.**

**Response:**

We sincerely thank the Reviewer for this important and insightful comment. We appreciate the opportunity to clarify the graphical representation and the statistical approach used to account for inter-patient variability.

The values presented in Figure 2 represent the observed median laboratory values with interquartile ranges (IQRs) at each predefined time point. These data were displayed as absolute values and were not normalized to baseline nor expressed as delta changes. As shown in Table 1, baseline laboratory parameters were comparable between groups; therefore, graphical baseline normalization was not applied.

Importantly, statistical comparisons were based on longitudinal linear mixed-effects models applied to the repeated individual-level laboratory measurements. The models included random intercepts to account for within-subject correlations and individual variability over time. This modeling framework appropriately accounts for inter-patient variability and repeated measurements without requiring baseline transformation in the graphical display. The Methods section has been revised accordingly.

**The revised** **Methods – Statistical Analysis** (page 9, paragraph 1, lines 196–198):

**Statistical Analysis**

All analyses were performed according to the per-protocol approach using R software (R Foundation for Statistical Computing, Vienna, Austria). An intention-to-treat analysis was not performed due to the minimal loss to follow-up and complete outcome data among randomized participants. Descriptive statistics were summarized as frequencies, means with standard deviations (SDs), or medians with interquartile ranges (IQRs), as appropriate. Between-group comparisons for categorical variables were performed using the chi-square test or Fisher’s exact test, as appropriate. Continuous variables were analyzed using independent *t*-tests or nonparametric equivalents when normality assumptions were not met. Longitudinal laboratory parameters were analyzed using linear mixed-effects models with random intercepts to account for within-subject correlations across repeated measurements. Maximum likelihood estimation was used for parameter estimation. For graphical presentation, observed median values with IQRs were displayed without baseline normalization or delta transformation. Inter-patient variability was addressed through the mixed-effects modeling framework.

**Comment 3: The introduction should provide more focused literature references regarding vitamin C’s effect on platelet counts in specific cancer types.**

**Response:**
We sincerely thank the Reviewer for this valuable and insightful comment. We agree that the Introduction should more specifically contextualize existing evidence regarding the effect of intravenous vitamin C (IVC) on platelet counts across different cancer types.

In response, we have revised the Introduction to incorporate more focused discussion of prior studies that evaluated IVC in relation to thrombocytopenia and platelet preservation in specific malignancies. We also clarified that findings across cancer types have been variable, thereby further justifying the need for investigation in nasopharyngeal carcinoma.

**The revised Introduction** (page 4, paragraph 1, lines 73–81):

Vitamin C (ascorbic acid) has garnered attention as a potential supportive therapy in oncology due to its antioxidant and anti-inflammatory properties. Since the 1970s, vitamin C has been investigated as a supportive treatment in oncology, with accumulating evidence suggesting its ability to alleviate chemotherapy-related symptoms such as nausea, vomiting, fatigue, insomnia, appetite loss, and cancer-related pain [10]. Moreover, intravenous vitamin C (IVC) has been explored for its potential role in mitigating chemotherapy-induced hematologic toxicities, particularly thrombocytopenia, through reduction of oxidative stress within the bone marrow microenvironment and preservation of megakaryocyte function [11,12]. For example, studies in ovarian cancer have reported reduced bone marrow toxicity and improved platelet recovery with adjunctive IVC administration [11], while investigations in hematologic malignancies have suggested potential restoration of hematopoietic function, including platelet counts [12]. However, findings across cancer types remain heterogeneous, and evidence in head and neck cancers, including nasopharyngeal carcinoma, is limited.

**Comment 4: The conclusion should be revised to emphasize the potential clinical benefit of IVC in mitigating platelet count reduction, which would better highlight the significance of this study.**

**Response:**
We sincerely thank the Reviewer for this insightful suggestion. We agree that the observed attenuation of platelet decline represents a potentially meaningful finding and should be more clearly emphasized in the Conclusion.

Accordingly, we have revised the Conclusion to highlight the association between IVC administration and preservation of platelet levels during chemoradiotherapy. We clarified that maintaining platelet stability may have implications for sustaining chemotherapy intensity and reducing the likelihood of bleeding or treatment interruptions. At the same time, we maintained a cautious and evidence-based tone to avoid overstating the findings.

**The revised Conclusion** (page 19, paragraph 2, lines 377-384):

**Conclusions**

IVC administration did not significantly reduce gastrointestinal adverse events in patients with nasopharyngeal carcinoma undergoing CCRT. However, it was associated with attenuation of platelet count decline during treatment. As this was a secondary outcome, preservation of platelet levels may help maintain treatment intensity and reduce the risk of bleeding or chemotherapy interruptions. This observation suggests a potential supportive effect of IVC on platelet-related toxicity during CCRT, although confirmatory studies are required.

**Response to Reviewer #4 comments**

**Comment:** This manuscript presents a well-designed randomized, double-blind, placebo-controlled clinical trial addressing an important supportive care question in nasopharyngeal carcinoma. The study is methodologically rigorous, with clear eligibility criteria, appropriate randomization and blinding procedures, and transparent reporting in line with CONSORT guidelines. The authors provide a balanced interpretation of both positive and null findings, particularly highlighting the clinically relevant observation of platelet preservation. Overall, the work adds meaningful evidence to the limited literature on intravenous vitamin C in this setting and is clearly written and well-structured. Hence this manuscript may be recommended.

**Response:**

We sincerely thank the Reviewer for the thoughtful, encouraging, and supportive evaluation of our manuscript. We greatly appreciate the recognition of the methodological rigor, adherence to CONSORT reporting standards, and the balanced interpretation of both significant and non-significant findings.

We are particularly grateful for the acknowledgment of the clinical relevance of platelet preservation and the contribution of this study to the limited literature on intravenous vitamin C in nasopharyngeal carcinoma. The Reviewer’s positive assessment underscores the importance of continued investigation in this area.

We thank the Reviewer again for the careful review and kind recommendation.

**Response to Reviewer #5 comments**

**Comment 1:** The primary endpoints are regarded as GI adverse events. So that when justifying the trial size of 22 per group (sensibly increased to 30), although power and test size are specified the anticipated difference between the groups is not. This difference should be specified.

**Response:**

We sincerely thank the Reviewer for this important and methodologically insightful comment. We agree that the anticipated effect size used in the sample size calculation should be explicitly stated for clarity and transparency.

In response, we have revised the Statistical Analysis section to specify the expected between-group difference used for sample size determination. The calculation was based on the proportions reported in Ma et al. [11], in which gastrointestinal adverse events occurred in 69% (9/13) of patients in the vitamin C group and 100% (12/12) in the control group, corresponding to an anticipated absolute risk reduction of approximately 31%. Using these proportions, with 80% power and a two-sided alpha of 0.05, the required sample size was estimated to be 22 participants per group. To account for potential attrition, additional participants were enrolled.

**The revised** **Statistical Analysis** (page 9, paragraph 2, lines 199-206):

# The sample size was calculated to detect a reduction in the incidence of gastrointestinal adverse events. The calculation was based on the proportions reported in a previous study by Ma et al. [11], in which gastrointestinal adverse events occurred in 9 of 13 patients (69%) in the vitamin C group and in 12 of 12 patients (100%) in the control group, corresponding to an anticipated absolute risk reduction of approximately 31%. Using these proportions, with 80% power and a two-sided alpha of 0.05, the required sample size was estimated to be 22 participants per group. To account for potential attrition, additional participants were enrolled.

# **Comment 2:** Table 1 Patient demographics: As this is a randomised trial then, by definition, any differences observed between characteristics in this table are ‘random’. So, testing statistical differences is wrong and the p-values in the table should be deleted.

**Response:**

We sincerely thank the Reviewer for this important methodological comment. We agree that formal hypothesis testing of baseline characteristics in randomized controlled trials is generally discouraged, as any observed differences arise from the randomization process and are expected to occur by chance rather than reflecting systematic bias.

In accordance with CONSORT recommendations, we have removed the p-values from Table 1 and now present baseline characteristics descriptively. The revised table reports summary statistics only, without statistical comparisons between groups.

**The revised** **Table 1** (page 11-12, lines 238-241):

**Table 1.** **Patient demographics.**

| **Patient characteristics** | **Vitamin C (n = 30)** | **Placebo (n = 30)** |
| --- | --- | --- |
| **Sex, n (%)** |  |  |
| Female | 8 (26.7) | 11 (36.7) |
| Male | 22 (73.3) | 19 (63.3) |
| **Age, mean ± SD (range)** | 50.3 ± 12.5 (20–71) | 50.3 ± 13.0 (20–73) |
| **Underlying disease, n (%)** |  |  |
| Hypertension | 6 (20.0) | 4 (13.3) |
| Diabetes mellitus | 2 (6.7) | 4 (13.3) |
| Dyslipidemia | 2 (6.7) | 3 (10.0) |
| Cardiovascular disease | 1 (3.3) | 1 (3.3) |
| Pulmonary disease | 0 (0.0) | 1 (3.3) |
| Hematologic disease | 0 (0.0) | 1 (3.3) |
| HIV | 0 (0.0) | 1 (3.3) |
| Others | 1 (3.3) | 1 (3.3) |
| **Stage, n (%)** |  |  |
| II | 3 (10.0) | 2 (6.7) |
| III | 9 (30.0) | 8 (26.7) |
| IVa | 18 (60.0) | 20 (66.7) |
| **Pretreatment laboratory values** |  |  |
| WBC (cells/µL), median (IQR) (range) | 7470 (5555–9580) (3350–21280) | 7070 (6230–8350) (4080–15280) |
| Hemoglobin (g/dL), mean ± SD (range) | 12.4 ± 1.9 (8.9–16.5) | 12.6 ± 1.7 (8.2–15.6) |
| Platelet (×10³ cells/µL), median (IQR) (range) | 312 (272–366) (143–649) | 306 (256–349) (211–644) |
| Creatinine (mg/dL), mean ± SD (range) | 0.8 ± 0.3 (0.40–1.62) | 0.9 ± 0.3 (0.55–1.68) |
| Sodium (mmol/L), mean ± SD (range) | 136.9 ± 3.2 (130.1–142.0) | 138.2 ± 3.2 (128.8–143.5) |
| Potassium (mmol/L), mean ± SD (range) | 3.9 ± 0.4 (3.07–5.00) | 3.9 ± 0.4 (2.75–5.19) |

Abbreviations: HIV, human immunodeficiency virus; SD, standard deviation; IQR, interquartile range; WBC, white blood cell

Continuous variables are presented as mean ± SD for approximately normally distributed data and as median (IQR) for non-normally distributed data.

**Comment 3:** Table 1: Probably better to describe minimum and maximum age rather than the SD. Also, for the pretreatment values WBC, Haemoglobin, etc.

**Response:**

We sincerely thank the Reviewer for this helpful suggestion. We agree that reporting the minimum and maximum values may provide additional clarity regarding the distribution of baseline characteristics.

In response, we have revised Table 1 to include the range (minimum–maximum) for age and all pretreatment laboratory parameters. Continuous variables are presented using mean ± standard deviation for approximately normally distributed variables and median (IQR) for non-normally distributed variables, with the range now provided to enhance descriptive transparency.

**The revised** **Table 1** (page 11-12, lines 238-241):

**Table 1.** **Patient demographics.**

| **Patient characteristics** | **Vitamin C (n = 30)** | **Placebo (n = 30)** |
| --- | --- | --- |
| **Sex, n (%)** |  |  |
| Female | 8 (26.7) | 11 (36.7) |
| Male | 22 (73.3) | 19 (63.3) |
| **Age, mean ± SD (range)** | 50.3 ± 12.5 (20–71) | 50.3 ± 13.0 (20–73) |
| **Underlying disease, n (%)** |  |  |
| Hypertension | 6 (20.0) | 4 (13.3) |
| Diabetes mellitus | 2 (6.7) | 4 (13.3) |
| Dyslipidemia | 2 (6.7) | 3 (10.0) |
| Cardiovascular disease | 1 (3.3) | 1 (3.3) |
| Pulmonary disease | 0 (0.0) | 1 (3.3) |
| Hematologic disease | 0 (0.0) | 1 (3.3) |
| HIV | 0 (0.0) | 1 (3.3) |
| Others | 1 (3.3) | 1 (3.3) |
| **Stage, n (%)** |  |  |
| II | 3 (10.0) | 2 (6.7) |
| III | 9 (30.0) | 8 (26.7) |
| IVa | 18 (60.0) | 20 (66.7) |
| **Pretreatment laboratory values** |  |  |
| WBC (cells/µL), median (IQR) (range) | 7470 (5555–9580) (3350–21280) | 7070 (6230–8350) (4080–15280) |
| Hemoglobin (g/dL), mean ± SD (range) | 12.4 ± 1.9 (8.9–16.5) | 12.6 ± 1.7 (8.2–15.6) |
| Platelet (×10³ cells/µL), median (IQR) (range) | 312 (272–366) (143–649) | 306 (256–349) (211–644) |
| Creatinine (mg/dL), mean ± SD (range) | 0.8 ± 0.3 (0.40–1.62) | 0.9 ± 0.3 (0.55–1.68) |
| Sodium (mmol/L), mean ± SD (range) | 136.9 ± 3.2 (130.1–142.0) | 138.2 ± 3.2 (128.8–143.5) |
| Potassium (mmol/L), mean ± SD (range) | 3.9 ± 0.4 (3.07–5.00) | 3.9 ± 0.4 (2.75–5.19) |

Abbreviations: HIV, human immunodeficiency virus; SD, standard deviation; IQR, interquartile range; WBC, white blood cell

Continuous variables are presented as mean ± SD for approximately normally distributed data and as median (IQR) for non-normally distributed data.

**Comment 4:** Table 2: For Nausea, the total of Grade 1 and Grade 2 adverse events with Vitamin C sums to greater than 30. So there so there is some double counting which makes the statistical testing invalid. The totals here should then be replaced by (say) the highest grade of adverse event experienced by the patient. The numbers experiencing zero adverse events should also be included to ensure that each sub table sums to 30 in each treatment group.

**Comment 5:** Table 2: In any event, although all may be incorrect because of the double counting, p-values are best quoted to 2 significant figures. So, 0.123 to 0.12 but ‘079’ remains as ‘0.079’, 0.155’ to 0.16’ and so on.
**Comment 6:** Table 2: Also, it would have helped to present, for example, for Oral mucositis (adding the zero group and presuming no double counting) the proportion of each Grade of adverse event in the margins of the sub tables. Here 7/25 = 0.28, 16/23 = 0.70, 3/3 = 0.5 and 4/6 = 0.67.

**Response:**

We sincerely thank the Reviewer for these careful and methodologically important observations regarding the presentation and statistical handling of Table 2. We appreciate the detailed suggestions, which have substantially improved the validity and clarity of our analysis.

**1. Double counting and patient-level analysis (Comment 4)**
We agree that the original presentation reflected cumulative event counts across chemotherapy cycles, which could potentially lead to double counting at the patient level. In accordance with the Reviewer’s recommendation, Table 2 has been fully revised to present the highest grade of each adverse event experienced per patient across the three chemotherapy cycles. This ensures that each patient contributes only once to each adverse event category.

Patients who did not experience the respective adverse event are now explicitly reported as “No adverse event,” ensuring that totals sum to 30 in each treatment group for every adverse event category. Statistical comparisons were recalculated using Fisher’s exact test based on these revised patient-level maximum grades.

Importantly, although p-values were recalculated using the corrected patient-level data, the overall findings remain unchanged. The incidence of gastrointestinal adverse events did not differ significantly between the vitamin C and placebo groups.

**2. P-value formatting (Comment 5)**
All p-values in Table 2 have been reformatted to two significant figures, in accordance with standard reporting conventions.

**3. Presentation of proportions (Comment 6)**
To enhance interpretability and internal consistency, Table 2 now reports both absolute numbers and corresponding percentages calculated within each treatment group (n = 30). This approach ensures that the proportion of each grade is clearly presented and that each adverse event category sums appropriately within groups.

The revised table is now entitled:

**“Table 2. Highest grades of chemotherapy-induced adverse effects observed in individual patients following three cycles of chemotherapy.”**

**The revised** **Table 2** (page 13, lines 253-255):

**Table 2. Highest grades of chemotherapy-induced adverse effects observed in individual patients following three cycles of chemotherapy.**

| **Adverse event grade** | **Vitamin C group**  **n/total (%)** | **Placebo group**  **n/total (%)** | ***p*-value** |
| --- | --- | --- | --- |
| Nausea |  |  | 0.23^§^ |
| No adverse event | 9/30 (30.00) | 15/30 (50.00) |  |
| Grade 1 | 17/30 (56.67) | 11/30 (36.67) |  |
| Grade 2 | 4/30 (13.33) | 4/30 (13.33) |  |
| Anorexia |  |  | 0.89^§^ |
| No adverse event | 10/30 (33.33) | 12/30 (40.00) |  |
| Grade 1 | 19/30 (63.33) | 17/30 (56.67) |  |
| Grade 2 | 1/30 (3.33) | 1/30 (3.33) |  |
| Oral mucositis |  |  | 1.00^§^ |
| No adverse event | 23/30 (76.67) | 23/30 (76.67) |  |
| Grade 1 | 0/30 (0.00) | 0/30 (0.00) |  |
| Grade 2 | 4/30 (13.33) | 4/30 (13.33) |  |
| Grade 3 | 3/30 (10.00) | 3/30 (10.00) |  |
| Diarrhea |  |  | 0.24^§^ |
| No adverse event | 27/30 (90.00) | 30/30 (100.00) |  |
| Grade 1 | 1/30 (3.33) | 0/30 (0.00) |  |
| Grade 2 | 2/30 (6.67) | 0/30 (0.00) |  |
| Dysphagia |  |  | 0.24^§^ |
| No adverse event | 27/30 (90.00) | 30/30 (100.00) |  |
| Grade 1 | 3/30 (10.00) | 0/30 (0.00) |  |
| Grade 2 | 0/30 (0.00) | 0/30 (0.00) |  |

^§^Fisher’s exact test

**Comment 7:** On a positive note, the authors when presenting results concerning the laboratory parameters refer to a Bayesian logistic regression. This is an unusual addition from a statistical perspective, but if retained needs much more explanation of what the methodology does and how the results are to be interpreted.

### ****Response:****

We sincerely thank the Reviewer for this insightful comment regarding the inclusion of Bayesian logistic regression analysis. We agree that the purpose and interpretation of the Bayesian approach should be more clearly explained to avoid potential ambiguity. The Bayesian analysis was included as a complementary exploratory method to provide a probabilistic interpretation of treatment effects, particularly given the exploratory nature of some secondary laboratory outcomes.

In response, we have expanded the Statistical Analysis section to clarify the rationale, methodology, and interpretation of the Bayesian models. Specifically, we now state that Bayesian logistic regression was applied to estimate the posterior probability that intravenous vitamin C was associated with changes in selected laboratory parameters. Weakly informative priors were used to avoid undue influence on the posterior estimates. Results are presented as the posterior probability (e.g., P(β < 0 | data)) is interpreted as the probability that the treatment effect is in a specified direction, given the observed data and prior assumptions.

**The revised** **Statistical Analysis** section (page 9, paragraph 2, lines 206–214):

In addition to frequentist analyses, Bayesian logistic regression models were fitted using weakly informative priors to estimate posterior probability distributions of treatment effects. This approach allows direct probabilistic interpretation of the direction and magnitude of treatment effects, which is particularly useful in the context of exploratory secondary outcomes. Results are summarized using posterior probabilities of benefit or harm. Treatment effects were interpreted according to whether the posterior distribution indicated a high probability of benefit or harm. The Bayesian analysis was included as a complementary exploratory framework to provide additional inferential insight.
